# Advances of Heat Shock Family in Ulcerative Colitis

**DOI:** 10.3389/fphar.2022.869930

**Published:** 2022-05-12

**Authors:** Min Gong, Fengrui Zhang, Yinglei Miao, Junkun Niu

**Affiliations:** ^1^ Department of Gastroenterology, The First Affiliated Hospital of Kunming Medical University, Kunming, China; ^2^ Yunnan Province Clinical Research Center for Digestive Diseases, Kunming, China

**Keywords:** ulcerative colitis, heat shock factor, heat shock protein, intestinal homeostasis, research advances

## Abstract

Ulcerative Colitis (UC) is a non-specific and chronic inflammatory disease of colonic mucosa whose exact etiology and mechanisms remain unclear. The incidence rate of UC is increasing year by year worldwide. What followed is that the medical costs are also rising rapidly. Therefore, it is urgent to understand the pathogenesis and find promising therapeutic targets for UC. Intestinal mucosal homeostasis is essential for normal bowel function, and its imbalance may be an important pathogenesis of UC. Endogenous homeostatic regulators play roles in repairing intestinal mucosa injury after stress. Heat shock family proteins are essential endogenous homeostasis factors. They can inhibit inflammation, regulate intestinal epithelial cells’ survival and death, and promote mucosal healing. Thus, they play important roles in sustaining intestinal mucosal homeostasis and protecting against UC progression. However, the heat shock family may promote UC carcinogenesis. Here, we summarize the advances in the research of the functions of the heat shock family in UC. And this review is an attempt to light on the etiopathogenesis of UC, highlighting the endogenous protective mechanisms, hoping to provide a novel therapeutic target for UC treatment.

## Overview of Ulcerative Colitis

Ulcerative colitis (UC) is an idiopathic, chronic inflammatory disorder of the colonic mucosa characterized by inflammatory ulceration of the colon and rectum. Patients with UC always present with bloody diarrhea, abdominal pain, and weight loss and are at increased risk of colorectal cancer (Lamb et al., 2019). Since this century, the incidence rate of ulcerative colitis has continued to rise (Ng et al., 2017), particularly in Asia, where the incidence was historically low (Ng et al., 2019). UC mainly occurs in young and middle-aged people, and the medical burden keeps growing (Alatab et al., 2020). Current studies show that the stimulation of environmental factors, intestinal flora, microorganisms, and antigens will induce excessive mucosal immune responses in genetically susceptible individuals, which is the cause of the onset of UC. However, the exact pathogenesis of UC remains unclear (Graham and Xavier, 2020), for which there is a lack of targeted pharmacotherapy medicine at present (Plichta et al., 2019). Biologic agents are only partially effective against partly patients, and their high cost and severe side effects limit their widespread use (Singh et al., 2018). Therefore, it is urgent to explore the pathogenesis and novel therapeutic target of UC.

## The Breakdown of Intestinal Mucosal Homeostasis is a Crucial Link in Ulcerative Colitis Occurrence and Development

**FIGURE 1:**
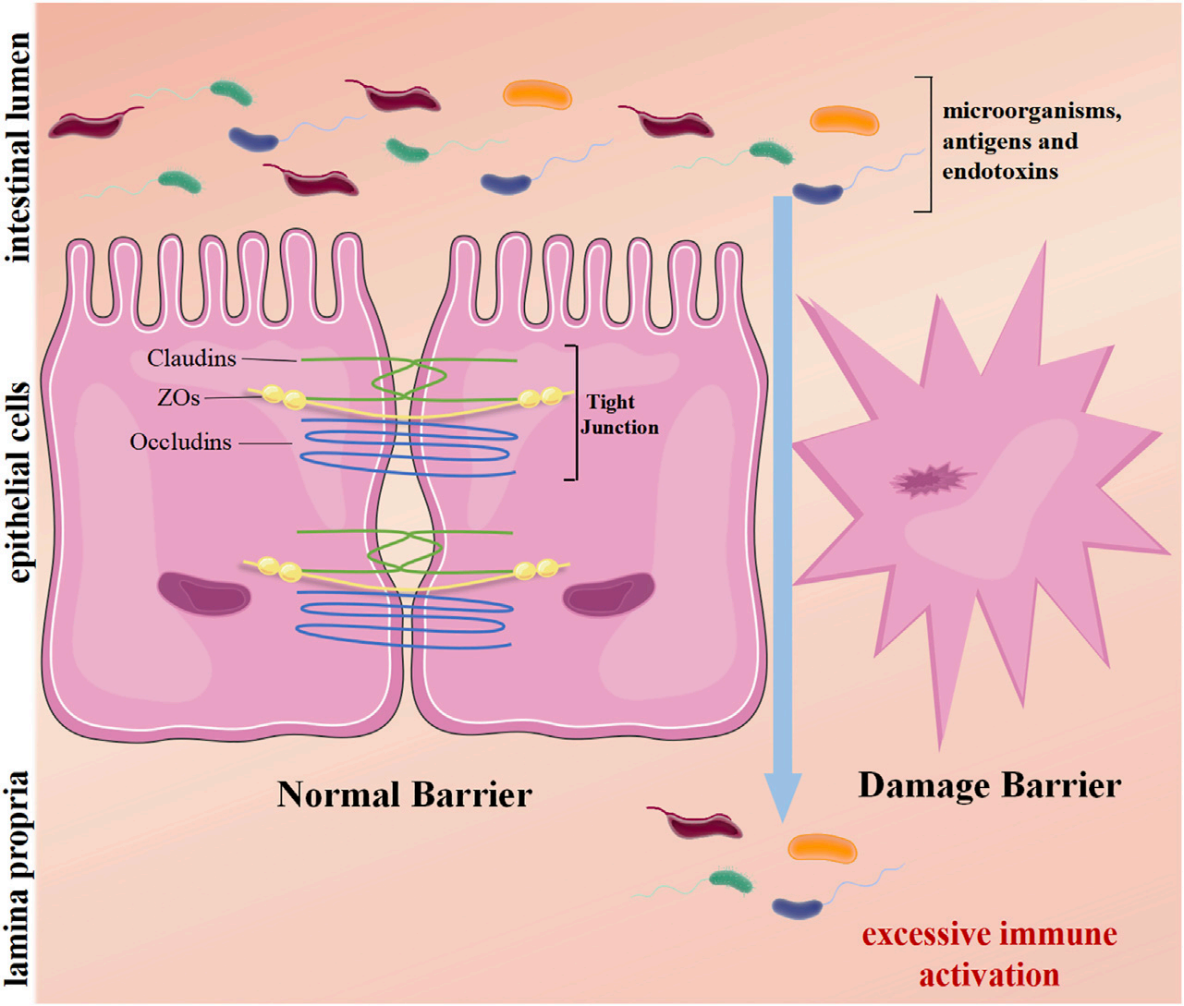
The mechanical barrier of the intestine. The intestinal epithelial cells and their tight junctions (TJs) form a physical barrier. TJs mainly consist of transmembrane proteins: occludins, claudins, and zonula occludens (ZOs). On average, harmful substances in the lumen cannot penetrate the epithelium. However, damage barriers allow toxic molecules to pass, induce inflammation, and excessive immune activation, causing intestinal disease.

Intestinal mucosal homeostasis ensures normal gut function, and its dyshomeostasis results in chronic intestinal inflammation (Maloy and Powrie, 2011). Intestinal homeostasis depends on the coregulation of multiple mechanisms. The integrity of the intestinal mucosal barrier is paramount. The intestinal mucosal barrier is composed of mechanical, chemical, immunological, and biological barriers, among which mechanical barriers play the most important role (Okumura and Takeda, 2018; Okumura and Takeda, 2017). Intestinal epithelial cells (IECs) and their tight junctions are the structural basis of the mechanical barrier. The tight junctions mainly contain three transmembrane proteins: Occludins, zonula occludens (ZOs), and Claudins (Buckley and Turner, 2018). This barrier can prevent excessive immune response by restraining harmful substances such as pathogenic microorganisms, antigens, and endotoxins from penetrating the upper cortex into the submucosa (Okumura and Takeda, 2018; Soderholm and Pedicord, 2019). Abnormalities of IECs or disruption of tight junctions may damage the mechanical barrier, giving rise to the occurrence and development of UC (Samoila et al., 2020) (Figure 1). Multiple studies support this view. Günther’s study found that IECs of UC patients had excessive apoptosis and necrosis in the period of disease activity, which triggered sustained inflammation (Günther et al., 2013). Kou’s research suggested that tight junction protein occludin expression in IECs of UC patients was lower than in healthy people, leading to the destruction of the intestinal mucosal mechanical barrier (Kuo et al., 2019). Hence, inhibiting excessive injury of IECs and maintaining the integrity of the intestinal mechanical wall may be potential targets of UC therapy. In the dyshomeostasis state, the expression of genes with endogenous intestinal protection is upregulated. Nowadays, numerous mucosa repair factors in UC, such as TFF (Aamann et al., 2014), TGF-β (Ihara et al., 2017), and EGF (Li et al., 2016), have been studied in detail. As a class of important endogenous protective factors *in vivo*, the heat shock protein family maintains the homeostasis of the intracellular environment and is closely related to various physiological and pathological conditions such as cell proliferation, death, and inflammation. The heat shock protein family has become a hot topic in maintaining intestinal mucosa homeostasis (Hoter and Naim, 2019).

## Physiological Functions of the Heat Shock Family

The heat shock family is a highly conserved family of molecules composed of six heat shock factors (HSF1-4, HSFX, and HSFY) and a wide diversity of heat shock proteins (HSPs) (Xu et al., 2012). HSF1,2, 4, HSFX, and HSFY are widely expressed in mammals, while HSF3 is only expressed in chickens and mice (Joutsen and Sistonen, 2019). Many studies have shown that HSFs are highly upregulated under the stressful environment of temperature rise and toxicity, and play critical roles in anti-stress, promoting growth, and maintaining the structure and function of cells (Akerfelt et al., 2010).

**FIGURE 2:**
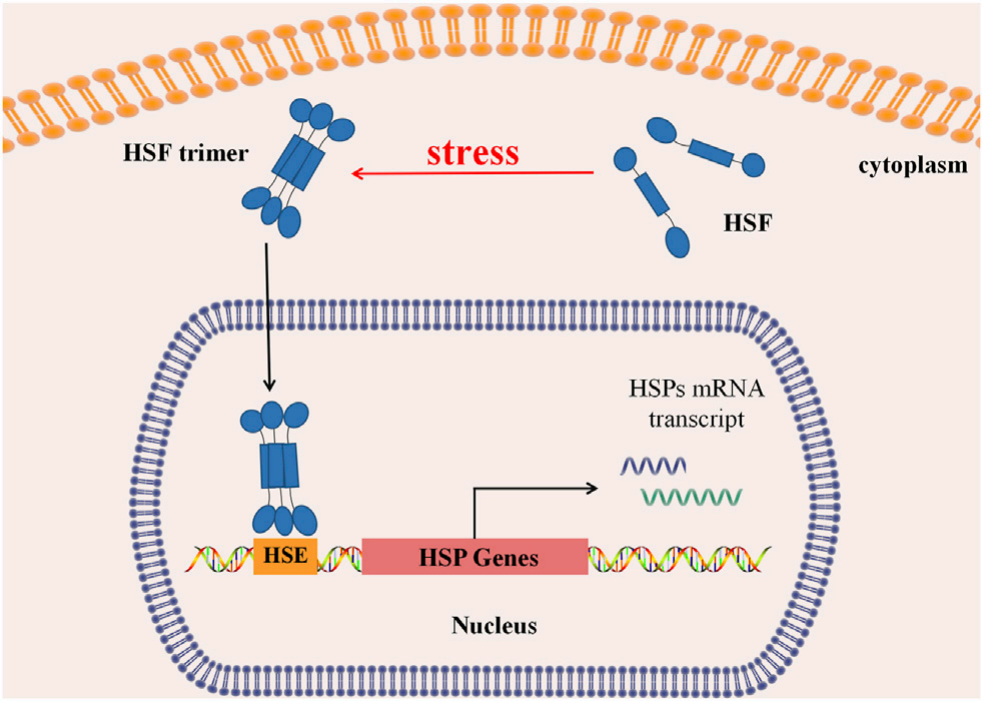
Regulation of heat shock response. In normal, HSFs stay in the cytoplasm as a monomer. Under stress, HSFs aggregate to form a trimer, which can be actively transported into the nucleus and binds with the Heat Shock regulatory element (HSE) to initiate downstream HSP transcription, causing Heat Shock Response (HSR), regulating transcription and expression of Heat Shock protein.

Under physiological conditions, deactivated HSFs are stored in the cytoplasm in monomers. When organisms are exposed to high temperatures or other stimuli such as heavy metals, bacteria, and bacterial toxins, monomer HSF polymerizes to form a homologous trimer, exposing DNA binding regions and nuclear localization sequences (NLS). Guided by NLS, HSF trimer transports into the nucleus *via* active transport. Then its DNA-biding part binds with heat shock regulatory element (HSE) to initiate transcription of downstream genes, induce heat shock response (HSR), and regulate HSP expression (Figure 2). When the stimuli factor disappears, the active HSF trimer depolymerizes to non-DNA-bound monomers, returning to the cytoplasm and nucleus, and the HSP transcription level returns to normal (Morimoto, 1993; Lang et al., 2021).

Studies have shown that HSF1 plays a major role in the human body and can work alone or combined with HSF2 (He et al., 2003). HSF1 is involved in diverse physiological and pathophysiological processes, such as cell cycle, apoptosis, and circadian rhythm (Kovács et al., 2019). It prevents cell death by protecting cells from protein toxicity (Gomez-Pastor et al., 2018), correlating with immunity (Shang et al., 2020), inflammation (Barber et al., 2014), tumor (Carpenter and Gökmen-Polar, 2019), and neurodegenerative diseases (Bose and Cho, 2017). HSF2 plays a synergistic role with HSF1 in regulating HSP expression under stress (Jaeger et al., 2016). It also takes part in mammalian growth and spermatogenesis (Akerfelt et al., 2007). HSF2 gene knock-out mice always have abnormal brain development or female sterility (Kallio et al., 2002). HSF4 is also indispensable in regulating lens development and maintaining the function of sensory organs (Akerfelt et al., 2007). Sex chromosome-related HSFX and HSFY are rarely understood, but they may be related to gametogenesis (Joutsen and Sistonen, 2019).

Heat shock protein (HSP) is a highly conservative stress-induced protein. Depending on their molecular weights, HSPs can be divided into six main families (HSP110, HSP90, HSP70, HSP60, HSP40, and sHSPs) (Jee, 2016). In addition, Kapinga proposed naming HSP members using letter/number combinations (Table 1) (Kampinga et al., 2009). HSP expression is regulated by HSF, which can protect cells from stress damage and prevent abnormal protein folding, and is involved in various autoimmune and chronic inflammatory diseases (Zininga et al., 2018).

**TABLE 1:** Heat shock protein family and its common members (Li et al., 2016).

HSP family	Alternative family name	Number of members	Common selected members
HSP110	HSPH	4	HSPH1 (HSP105), HSPH1(HSP110, HSPA4)
HSP90	HSPC	5	HSPC2 (HSP90α), HSPC3(HSP90β), HSPC4 (GRP94,HSP90B1, GP96, endoplasmin), HSPC5 (TRAP1, HSP75, HSP90L)
HSP70	HSPA	13	HSPA1A (HSP70-1), HSPA1B (HSP70-2) HSPA5 (BIP, GRP78), HSPA6 (HSP70B0), HSPA8 (HSC70), HSPA9 (GRP75)
HSP60、HSP10 (Chaperonins)	HSPD、HSPE	14	HSPD1 (HSP60), HSPE1 (HSP10)
HSP40	DNAJ	50	DNAJA1, DNAJB1 (HSPF1 and HSP40), DNAJC1
Small HSPs	HSPB	11	HSPB1 (HSP27), HSPB4 (CRYAA) and HSPB5 (CRYAB)

Recent studies have found that the heat shock family plays an essential role in intestinal inflammation-associated disease and is proposed as a crucial endogenous protecting factor in maintaining mucosal homeostasis (Finlayson-Trick et al., 2018; Zhang et al., 2021). Tenaka’s study indicated that disease activity, colon epithelial cells (CECs) apoptosis rate, and mucosal damage level of HSF1 overexpressed transgenic mice were significantly reduced compared with wild-type mice in DSS-induced mice colitis models (Tanaka et al., 2007). This study confirmed that HSF1 is a protective factor for colitis at the genetic level. This protection may involve the downregulation of pro-inflammatory cytokine expressions such as IL-1b, IL-6, and TNF-a and inhibition of ROS-induced cell death (Tanaka et al., 2007). These findings suggest that the heat shock family can be highly relevant in ulcerative colitis development. Therefore, induction of specific heat shock family members may be a new therapeutic target for UC.

## The Role of Heat Shock Family in Maintaining Ulcerative Colitis Mucosal Homeostasis

### The Heat Shock Family Maintains Mucosal Homeostasis by Inhibiting Intestinal Inflammation

In a physiological state, HSFs and HSPs are continuously expressed in the epithelium of the colon mucosa because of the specific substances in the colonic luminal environment, such as intestinal flora, lipopolysaccharides (LPS), and short-chain fatty acids (SCFAs) (Wells et al., 2017). For the persistent inflammation in UC patients, an experiment conducted by Robert’s team found that HSF1 knock-out mice secreted more TNF-a, IL-1, IL-6, and IL-6 and had a more intense inflammatory response than wild-type mice (Barber et al., 2014). Coincidentally, a study of DSS-induced colitis in mice designed by Zhang and his team found that HSF2 knock-out mice also had more severe intestinal inflammation than wild-type mice (Zhang et al., 2020a). This evidence suggests that the heat shock family significantly correlates with intestinal inflammation. Knowlton’s study showed that HSF1 can downregulate TNF-a and IL-1b transcription by binding to TNF -a promoter and IL-1b transcription factors and directly inhibit the expression of NF-κB and nuclear transcription factor activator protein-1 (AP-1) from alleviating inflammation (Knowlton, 2006). Because of highly homologous with HSF1, HSF2 has been confirmed to have a similar function as HSF1. HSF2 could reduce IL-1b secretion by suppressing NLRP3 inflammasome activation (Zhang et al., 2021) or regulating the mitogen-activated protein kinase (MAPK) pathway (Wen et al., 2020) to act as an anti-inflammatory.

Studies on HSPs mainly focus on a few HSPs, such as HSP70 and HSP27. Wang and his team used four different experimental colitis models to identify two distinct protective functions for Hsp70: promoting intestinal homeostasis by interaction with ZO-1 to stabilize tight junctions and limiting inflammatory-mediated mucosal damage by affecting ERK phosphorylation and regulating IL-10 production in immune cells (Wang et al., 2018). Additionally, HSP70 can also downregulate the production of inflammatory factors such as TNF-a and IFN-γ by interplay with dendritic cells (DC) and monocytes (Borges et al., 2012). Another experiment detected inflammatory factors expression levels in LPS-stimulated cells pretreated by HSP27 specific phosphorylation inhibitors, found that phosphorylated HSP27 can increase the level of IKB-a by inhibiting the phosphorylation level of IkB-a (pIkB-a) and then restrain the NF-kB pathway to play a protective role in inflammation (Chen and Currie, 2006; Zhang et al., 2020b).

### Heat Shock Family Maintains Mucosal Homeostasis by Regulating the Survival and Death of Intestinal Epithelial Cells and Promoting Mucosal Healing

Intestinal epithelial cells (IECs), as a critical factor of intestinal homeostasis, are the essential structural basis of the mechanical barrier. They have multiple functions such as nutrient absorption, antimicrobial peptides secretion, immune response regulation, and separation of intestinal microbial flora (Soderholm and Pedicord, 2019). When the intestinal mucosal epithelium is injured, the body restores the intestinal mechanical barrier by regulating the proliferation and differentiation of intestinal epithelial cells and the interaction between different intestinal immune cells (such as intestinal macrophages, granulocytes, and lymphocytes). The heat shock family is widely involved in these processes. Studies have shown that HSF1 inhibits the NF-kB signaling pathway, downregulates NLRP3 inflammasome and caspase-1 production, and restrains IECs apoptosis by regulating Toll-like receptors (TLRs) expression (Shang et al., 2020; Saber and El-Kader, 2021). HSF2 and HSP27 regulate the IECs apoptosis, resulting from their regulation of mitochondrial pathway and restriction of reactive oxygen species (ROS), respectively (Xie et al., 2016; Wang et al., 2020).

In UC, excessive apoptosis occurs in IECs, leading to pyroptosis (Ey et al., 2013) and ferroptosis (Xu et al., 2020). That impairs the mechanical barrier and makes the disease worse. HSF1 and HSF2 can inhibit ROS production by promoting HSP70 expression (Wilkerson et al., 2007; Wu et al., 2013). They can downregulate pyroptosis (Zhou et al., 2020) and ferroptosis (Song et al., 2019; Chen et al., 2020) in intestinal epithelial cells to relieve UC inflammation. On the other hand, autophagy plays an essential role in protecting IECs from damage (Lassen and Xavier, 2018) and maintaining mucosal barrier and homeostasis (Foerster et al., 2022). Studies have suggested that some HSF70 family members are associated with autophagy, and HSF1 and HSF2 can regulate autophagy activity indirectly by attending to the accommodation of HSP70 expression, which maintains intestinal mucosal homeostasis (Borges et al., 2012).

Moreover, HSF1,2 can regulate the TGF-β/Smad signaling pathway (Wen et al., 2020), promote the proliferation and differentiation of colon crypt stem cells, and replace senescent and necrotic IECs (Miyoshi et al., 2012), which can facilitate intestinal tissue remodeling (Biancheri et al., 2014). Chu and his team studied the CCD-18CO human colonic myofibroblast cell line. This study found that HSP27 accelerates wound healing by regulating colonic myofibroblast migration (Chu et al., 2017). The above finding proved that the heat shock family plays a positive role in repairing colonic mucosal damage in UC patients.

These shreds of evidence demonstrate that the heat shock family plays a protective role in inhibiting the inflammatory response process of UC, maintaining the integrity of the intestinal mucosal barrier, and promoting the reconstruction of mucosal. However, other vital regulatory factors and specific targets of heat shock family protection of IECs still need intensive study. Further elucidation of the regulatory mechanism of endogenous homeostasis factors in the regulation of intestinal homeostasis could provide new clues for exploiting novel therapies of UC.

## The Role of the Heat Shock Family in the Occurrence and Development of Colorectal Cancer

Colorectal Cancer (CRC) is the 3rd most crucial cancer globally, ranking 4th mortality, accounting for approximately 10% of cancer-related deaths (Dekker et al., 2019). Most colorectal cancers development follows the polyps (precancerous lesions)-adenomas (polyps)-colorectal cancer model (Dekker et al., 2019). Many studies have shown that the heat shock family is highly associated with CRC. Ren et al., (2022)’s bioinformatics study on CRC found that HSF1 expression in colorectal cancer presents a noticeable increasing tendency. Its expression level was correlated to the tumor stage and extent of lymph node metastasis. An experiment that used an AOM/DSS-induced colorectal cancer model found that HSF1 inhibited the expression of microRNA137 (MIR137) targeting glutaminase 1 (GLS1) through DNMT3a recruitment, stimulated the activation of GLS1-dependent mTOR, and promoted colorectal cancer (Li et al., 2018). In addition, HSF1 has been found to maintain high expression of Dickkopf-3 (DKK3) in the matrix by interaction with the DKK3 promoter and enhancer, enhance the Wnt signaling pathway, inhibit YAP/TAZ degradation, and promote tumor invasion (Ferrari et al., 2019). Another study based on The Cancer Genome Atlas-Colorectal Cancer (TCGA-CRC) also found that HSF4-mRNA expression increased in CRC tissues, and the proportion of stage III/IV CRC in patients with high HSF4 expression was much higher than that in patients with low ones (Yang et al., 2017). A multivariate regression analysis of 297 CRC patients revealed that patients with HSP70 upregulation had a poor prognosis (shorter disease-free survival and poor tumor differentiation) (Hrudka et al., 2021). Cen and his team also found that, compared with normal, the increased expression of HSP27 and HSP90 can be observed in CRC tissues (Cen et al., 2004).

Ulcerative Colitis-Associated Colorectal Cancer (CAC) is one of the most life-threatening consequences of chronic ulcerative colitis (Yashiro, 2014). Unlike sporadic CRC, CAC does not show the characteristic pathophysiological process (adenoma-carcinogenesis) but presents as chronic inflammation →epithelial cell migration →atypical hyperplasia →CAC. Current studies suggest that chronic inflammatory stress, such as ROS and some radicals, is the leading cause of dysplasia (Rogler, 2014). In this regard, the heat shock family may inhibit the progression of UC to CAC under its function, including maintaining internal environment stability and resisting oxidative stress. However, a study by Oshrat and his team on colitis-associated colon cancer (CAC) convinced that HSF1 regulates extracellular matrix (ECM) remodeling in mouse colon fibroblasts to promote tumor progression by upregulating the transcription of genes encoding matrix proteins (FN1 and LAMA1), matrix remodeling enzymes (MMP7 and MMP9), and HSP47 (Levi-Galibov et al., 2020). In other words, the increased expression of HSF1 may promote the occurrence of ulcerative colitis-associated colon cancer. These studies suggest that the heat shock family has a dual role in different disease stages of UC. Therefore, further exploration of the role and mechanism of the heat shock family in other disease processes is of great guiding significance for using heat shock family members as new targets for UC treatment.

## Heat Shock Family Could be an Indicator of the Treatment Effectiveness Assessment of Ulcerative Colitis

Mucosal healing (MH) refers to the process in which epithelial cells near the deficient mucosa recover, proliferate, differentiate and cover the defect surface, and then re-establish mucosal homeostasis (Iizuka and Konno, 2011), which is the therapeutic goal of ulcerative colitis (Ungaro et al., 2019). Achieving and maintaining long-term mucosal healing reduces the risk of relapse, colectomy, and UC-associated colon cancer (Boal Carvalho et al., 2016). Endoscopy is the foremost approach to evaluate mucosal healing, among which Mayo endoscopy score (MES) is the most widely used in clinical practice (Moriichi et al., 2021). However, repeated endoscopic examination is expensive and invasive. Besides, it is difficult for endoscopy to reflect submucosal conditions accurately. Several studies have shown that there is still active histological inflammation during mucosal healing under endoscopy (Moriichi et al., 2021). Fecal Calprotectin (FC), the other marker used to evaluate MH, has a particular value in predicting MH. Nevertheless, samples significantly affect their value, and the cut-off value used to assess MH in various studies also has significant differences (Ricciuto and Griffiths, 2019; Malvão et al., 2021). Therefore, it is a potential research direction to explore new biomarkers from intestinal mucosal homeostasis protective factors to make up for the deficiency of endoscopy and FC in MH assessment.

Multiple HSF and HSP are involved in the MH process, and their expression changes reflect mucosal injury repair status. A report indicated that compared with healthy people, the expression of diversified HSP (Rodolico et al., 2010) and HSF (Miao et al., 2013) are different in UC patients, and such expression differences exist in serum, colonic mucosa, and feces (Zhang et al., 2020a). More importantly, HSF2 expression was positively correlated with the severity of UC (Miao et al., 2014). In a single-center study, fecal HSF2 quantity was found to predict MH with a sensitivity of 73.7% and specificity of 70.1%. Although its sensitivity and specificity were lower than FC (84.2% and 79.9%) (Wen et al., 2020), it was still a valuable explorative study on the heat shock family in assessing the efficacy of UC. Furthermore, Tomasello’s study convinced that the expression levels of HSP10, HSP70, and HSP90 in the mucosa of active UC patients decreased strongly after therapy (Tomasello et al., 2011). These results suggest that the heat shock family is promising as a new endogenous biomarker for evaluating the degree of inflammatory activity in UC.

## Conclusion and Prospect

The pathogenesis of UC is still unclear, and colonic mucosal homeostasis is on the cutting edge of UC etiology research. The dynamic balance between mucosal injury and repair is the key to maintaining mucosal homeostasis. Previous research mainly concentrated on the effect and mechanism of the inflammatory signaling pathway in mucosal excessive immune injury. Nevertheless, the body inhibits the exaggerated inflammatory response and epithelial cell damage while promoting mucosal repair factors and epithelial cell renewal. The function of the mucosal mechanical barrier is crucial to intestinal homeostasis, and the dynamic balance of IECs loss and self-renewal is the key to maintaining it. Endogenous protective factors are essential in sustaining mucosal homeostasis, and their effect on UC is a novel research orientation. The heat shock family is crucial for inhibiting inflammation, promoting mucosal repair, and promoting the transition from UC to CAC. In-depth exploration of the role of the heat shock family in various disease stages of UC, especially in maintaining intestinal mucosal homeostasis, may provide a brand new perspective and theory for annotating the mechanical barrier function of UC mucosa and developing therapeutic targets.

## References

[B1] Aamann L. Vestergaard E. M. Grønbæk H. (2014). Trefoil Factors in Inflammatory Bowel Disease. World J. Gastroenterol. 20 (12), 3223–3230. 10.3748/wjg.v20.i12.3223 24696606 PMC3964394

[B2] Akerfelt M. Morimoto R. I. Sistonen L. (2010). Heat Shock Factors: Integrators of Cell Stress, Development and Lifespan. Nat. Rev. Mol. Cell Biol. 11 (8), 545–555. 10.1038/nrm2938 20628411 PMC3402356

[B3] Akerfelt M. Trouillet D. Mezger V. Sistonen L. (2007). Heat Shock Factors at a Crossroad between Stress and Development. Ann. N. Y. Acad. Sci. 1113, 15–27. 10.1196/annals.1391.005 17483205

[B4] Alatab S. Sepanlou S. G. Ikuta K. Vahedi H. Bisignano C. Safiri S. (2020). The Global, Regional, and National Burden of Inflammatory Bowel Disease in 195 Countries and Territories, 1990-2017: a Systematic Analysis for the Global Burden of Disease Study 2017. Lancet Gastroenterol. Hepatol. 5 (1), 17–30. 10.1016/S2468-1253(19)30333-4 31648971 PMC7026709

[B5] Barber R. C. Maass D. L. White D. J. Horton J. W. Wolf S. E. Minei J. P. (2014). Deficiency in Heat Shock Factor 1 (HSF-1) Expression Exacerbates Sepsis-Induced Inflammation and Cardiac Dysfunction. SOJ Surg. 1 (1). 10.15226/2376-4570/1/1/00103 PMC6349382 30701190

[B6] Biancheri P. Giuffrida P. Docena G. H. MacDonald T. T. Corazza G. R. Di Sabatino A. (2014). The Role of Transforming Growth Factor (TGF)-β in Modulating the Immune Response and Fibrogenesis in the Gut. Cytokine Growth Factor Rev. 25 (1), 45–55. 10.1016/j.cytogfr.2013.11.001 24332927

[B7] Boal Carvalho P. Dias de Castro F. Rosa B. Moreira M. J. Cotter J. (2016). Mucosal Healing in Ulcerative Colitis--When Zero Is Better. J. Crohns Colitis 10 (1), 20–25. 10.1093/ecco-jcc/jjv180 26438714

[B8] Borges T. J. Wieten L. van Herwijnen M. J. Broere F. van der Zee R. Bonorino C. (2012). The Anti-inflammatory Mechanisms of Hsp70. Front. Immunol. 3, 95. 10.3389/fimmu.2012.00095 22566973 PMC3343630

[B9] Bose S. Cho J. (2017). Targeting Chaperones, Heat Shock Factor-1, and Unfolded Protein Response: Promising Therapeutic Approaches for Neurodegenerative Disorders. Ageing Res. Rev. 35, 155–175. 10.1016/j.arr.2016.09.004 27702699

[B10] Buckley A. Turner J. R. (2018). Cell Biology of Tight Junction Barrier Regulation and Mucosal Disease. Cold Spring Harb. Perspect. Biol. 10 (1). 10.1101/cshperspect.a029314 PMC5749156 28507021

[B11] Carpenter R. L. Gökmen-Polar Y. (2019). HSF1 as a Cancer Biomarker and Therapeutic Target. Curr. Cancer Drug Targets 19 (7), 515–524. 10.2174/1568009618666181018162117 30338738 PMC6472998

[B12] Cen H. Zheng S. Fang Y. M. Tang X. P. Dong Q. (2004). Induction of HSF1 Expression Is Associated with Sporadic Colorectal Cancer. World J. Gastroenterol. 10 (21), 3122–3126. 10.3748/wjg.v10.i21.3122 15457556 PMC4611254

[B13] Chen Y. Currie R. W. (2006). Small Interfering RNA Knocks Down Heat Shock Factor-1 (HSF-1) and Exacerbates Pro-inflammatory Activation of NF-kappaB and AP-1 in Vascular Smooth Muscle Cells. Cardiovasc Res. 69 (1), 66–75. 10.1016/j.cardiores.2005.07.004 16061216

[B14] Chen Y. Zhang P. Chen W. Chen G. (2020). Ferroptosis Mediated DSS-Induced Ulcerative Colitis Associated with Nrf2/HO-1 Signaling Pathway. Immunol. Lett. 225, 9–15. 10.1016/j.imlet.2020.06.005 32540488

[B15] Chu E. Saini S. Liu T. Yoo J. (2017). Bradykinin Stimulates Protein Kinase D-Mediated Colonic Myofibroblast Migration via Cyclooxygenase-2 and Heat Shock Protein 27. J. Surg. Res. 209, 191–198. 10.1016/j.jss.2016.10.014 28032559 PMC5393353

[B16] Dekker E. Tanis P. J. Vleugels J. L. A. Kasi P. M. Wallace M. B. (2019). Colorectal Cancer. Lancet 394 (10207), 1467–1480. 10.1016/S0140-6736(19)32319-0 31631858

[B17] Ey B. Eyking A. Klepak M. Salzman N. H. Göthert J. R. Rünzi M. (2013). Loss of TLR2 Worsens Spontaneous Colitis in MDR1A Deficiency through Commensally Induced Pyroptosis. J. Immunol. 190 (11), 5676–5688. 10.4049/jimmunol.1201592 23636052 PMC3659955

[B18] Ferrari N. Ranftl R. Chicherova I. Slaven N. D. Moeendarbary E. Farrugia A. J. (2019). Dickkopf-3 Links HSF1 and YAP/TAZ Signalling to Control Aggressive Behaviours in Cancer-Associated Fibroblasts. Nat. Commun. 10 (1), 130. 10.1038/s41467-018-07987-0 30631061 PMC6328607

[B19] Finlayson-Trick E. Connors J. Stadnyk A. Van Limbergen J. (2018). Regulation of Antimicrobial Pathways by Endogenous Heat Shock Proteins in Gastrointestinal Disorders. GastrointestDisord 1 (1), 39–56. 10.3390/gidisord1010005

[B20] Foerster E. G. Mukherjee T. Cabral-Fernandes L. Rocha J. D. B. Girardin S. E. Philpott D. J. (2022). How Autophagy Controls the Intestinal Epithelial Barrier. Autophagy 18 (1), 86–103. 10.1080/15548627.2021.1909406 33906557 PMC8865220

[B21] Gomez-Pastor R. Burchfiel E. T. Thiele D. J. (2018). Regulation of Heat Shock Transcription Factors and Their Roles in Physiology and Disease. Nat. Rev. Mol. Cell Biol. 19 (1), 4–19. 10.1038/nrm.2017.73 28852220 PMC5794010

[B22] Graham D. B. Xavier R. J. (2020). Pathway Paradigms Revealed from the Genetics of Inflammatory Bowel Disease. Nature 578 (7796), 527–539. 10.1038/s41586-020-2025-2 32103191 PMC7871366

[B23] Günther C. Neumann H. Neurath M. F. Becker C. (2013). Apoptosis, Necrosis and Necroptosis: Cell Death Regulation in the Intestinal Epithelium. Gut 62 (7), 1062–1071. 10.1136/gutjnl-2011-301364 22689519

[B24] He H. Soncin F. Grammatikakis N. Li Y. Siganou A. Gong J. (2003). Elevated Expression of Heat Shock Factor (HSF) 2A Stimulates HSF1-Induced Transcription during Stress. J. Biol. Chem. 278 (37), 35465–35475. 10.1074/jbc.M304663200 12813038

[B25] Hoter A. Naim H. Y. (2019). The Functions and Therapeutic Potential of Heat Shock Proteins in Inflammatory Bowel Disease-An Update. Int. J. Mol. Sci. 20 (21). 10.3390/ijms20215331 PMC6862201 31717769

[B26] Hrudka J. Jelínková K. Fišerová H. Matĕj, K. Mandys V. Waldauf P. (2021). Heat Shock Proteins 27, 70, and 110: Expression and Prognostic Significance in Colorectal Cancer. Cancers (Basel). 13 (17), 4407. 10.3390/cancers13174407 34503216 PMC8431468

[B27] Ihara S. Hirata Y. Koike K. (2017). TGF-β in Inflammatory Bowel Disease: a Key Regulator of Immune Cells, Epithelium, and the Intestinal Microbiota. J. Gastroenterol. 52 (7), 777–787. 10.1007/s00535-017-1350-1 28534191

[B28] Iizuka M. Konno S. (2011). Wound Healing of Intestinal Epithelial Cells. World J. Gastroenterol. 17 (17), 2161–2171. 10.3748/wjg.v17.i17.2161 21633524 PMC3092866

[B29] Jaeger A. M. Pemble C. W. Sistonen L. Thiele D. J. (2016). Structures of HSF2 Reveal Mechanisms for Differential Regulation of Human Heat-Shock Factors. Nat. Struct. Mol. Biol. 23 (2), 147–154. 10.1038/nsmb.3150 26727490 PMC4973471

[B30] Jee H. (2016). Size Dependent Classification of Heat Shock Proteins: a Mini-Review. J. Exerc Rehabil. 12 (4), 255–259. 10.12965/jer.1632642.321 27656620 PMC5031383

[B31] Joutsen J. Sistonen L. (2019). Tailoring of Proteostasis Networks with Heat Shock Factors. Cold Spring Harb. Perspect. Biol. 11 (4). 10.1101/cshperspect.a034066 PMC6442201 30420555

[B32] Kallio M. Chang Y. Manuel M. Alastalo T. P. Rallu M. Gitton Y. (2002). Brain Abnormalities, Defective Meiotic Chromosome Synapsis and Female Subfertility in HSF2 Null Mice. EMBO J. 21 (11), 2591–2601. 10.1093/emboj/21.11.2591 12032072 PMC125382

[B33] Kampinga H. H. Hageman J. Vos M. J. Kubota H. Tanguay R. M. Bruford E. A. (2009). Guidelines for the Nomenclature of the Human Heat Shock Proteins. Cell Stress Chaperones 14 (1), 105–111. 10.1007/s12192-008-0068-7 18663603 PMC2673902

[B34] Knowlton A. A. (2006). NFkappaB, Heat Shock Proteins, HSF-1, and Inflammation. Cardiovasc Res. 69 (1), 7–8. 10.1016/j.cardiores.2005.10.009 16337613

[B35] Kovács D. Sigmond T. Hotzi B. Bohár B. Fazekas D. Deák V. (2019). HSF1Base: A Comprehensive Database of HSF1 (Heat Shock Factor 1) Target Genes. Int. J. Mol. Sci. 20 (22). 10.3390/ijms20225815 PMC6888953 31752429

[B36] Kuo W. T. Shen L. Zuo L. Shashikanth N. Ong M. L. D. M. Wu L. (2019). Inflammation-induced Occludin Downregulation Limits Epithelial Apoptosis by Suppressing Caspase-3 Expression. Gastroenterology 157 (5), 1323–1337. 10.1053/j.gastro.2019.07.058 31401143 PMC6815722

[B37] Lamb C. A. Kennedy N. A. Raine T. Hendy P. A. Smith P. J. Limdi J. K. (2019). British Society of Gastroenterology Consensus Guidelines on the Management of Inflammatory Bowel Disease in Adults. Gut 68 (Suppl. 3), s1–s106. 10.1136/gutjnl-2019-318484 31562236 PMC6872448

[B38] Lang B. J. Guerrero M. E. Prince T. L. Okusha Y. Bonorino C. Calderwood S. K. (2021). The Functions and Regulation of Heat Shock Proteins; Key Orchestrators of Proteostasis and the Heat Shock Response. Arch. Toxicol. 95 (6), 1943–1970. 10.1007/s00204-021-03070-8 34003342

[B39] Lassen K. G. Xavier R. J. (2018). Mechanisms and Function of Autophagy in Intestinal Disease. Autophagy 14 (2), 216–220. 10.1080/15548627.2017.1389358 29130415 PMC5902220

[B40] Levi-Galibov O. Lavon H. Wassermann-Dozorets R. Pevsner-Fischer M. Mayer S. Wershof E. (2020). Heat Shock Factor 1-dependent Extracellular Matrix Remodeling Mediates the Transition from Chronic Intestinal Inflammation to Colon Cancer. Nat. Commun. 11 (1), 6245. 10.1038/s41467-020-20054-x 33288768 PMC7721883

[B41] Li J. Song P. Jiang T. Dai D. Wang H. Sun J. (2018). Heat Shock Factor 1 Epigenetically Stimulates Glutaminase-1-dependent mTOR Activation to Promote Colorectal Carcinogenesis. Mol. Ther. 26 (7), 1828–1839. 10.1016/j.ymthe.2018.04.014 29730197 PMC6035735

[B42] Li J. Wei Z. Chang X. Cardinale C. J. Kim C. E. Baldassano R. N. (2016). Pathway-based Genome-wide Association Studies Reveal the Association between Growth Factor Activity and Inflammatory Bowel Disease. Inflamm. Bowel Dis. 22 (7), 1540–1551. 10.1097/MIB.0000000000000785 27104816

[B43] Maloy K. J. Powrie F. (2011). Intestinal Homeostasis and its Breakdown in Inflammatory Bowel Disease. Nature 474 (7351), 298–306. 10.1038/nature10208 21677746

[B44] Malvão L. D. R. Madi K. Esberard B. C. de Amorim R. F. Silva K. D. S. Farias E Silva K. (2021). Fecal Calprotectin as a Noninvasive Test to Predict Deep Remission in Patients with Ulcerative Colitis. Med. Baltim. 100 (3), e24058. 10.1097/MD.0000000000024058 PMC7837839 33546007

[B45] Miao J. Niu J. Wang K. Xiao Y. Du Y. Zhou L. (2014). Heat Shock Factor 2 Levels Are Associated with the Severity of Ulcerative Colitis. PLoS One 9 (2), e88822. 10.1371/journal.pone.0088822 24533153 PMC3923051

[B46] Miao Y. L. Xiao Y. L. Du Y. Duan L. P. (2013). Gene Expression Profiles in Peripheral Blood Mononuclear Cells of Ulcerative Colitis Patients. World J. Gastroenterol. 19 (21), 3339–3346. 10.3748/wjg.v19.i21.3339 23745037 PMC3671087

[B47] Miyoshi H. Ajima R. Luo C. T. Yamaguchi T. P. Stappenbeck T. S. (2012). Wnt5a Potentiates TGF-β Signaling to Promote Colonic Crypt Regeneration after Tissue Injury. Science 338 (6103), 108–113. 10.1126/science.1223821 22956684 PMC3706630

[B48] Moriichi K. Fujiya M. Okumura T. (2021). The Endoscopic Diagnosis of Mucosal Healing and Deep Remission in Inflammatory Bowel Disease. Dig. Endosc. 33 (7), 1008–1023. 10.1111/den.13863 33020947

[B49] Morimoto R. I. (1993). Cells in Stress: Transcriptional Activation of Heat Shock Genes. Science 259 (5100), 1409–1410. 10.1126/science.8451637 8451637

[B50] Ng S. C. Kaplan G. G. Tang W. Banerjee R. Adigopula B. Underwood F. E. (2019). Population Density and Risk of Inflammatory Bowel Disease: A Prospective Population-Based Study in 13 Countries or Regions in Asia-Pacific. Am. J. Gastroenterol. 114 (1), 107–115. 10.1038/s41395-018-0233-2 30177785

[B51] Ng S. C. Shi H. Y. Hamidi N. Underwood F. E. Tang W. Benchimol E. I. (2017). Worldwide Incidence and Prevalence of Inflammatory Bowel Disease in the 21st Century: a Systematic Review of Population-Based Studies. Lancet 390 (10114), 2769–2778. 10.1016/S0140-6736(17)32448-0 29050646

[B52] Okumura R. Takeda K. (2018). Maintenance of Intestinal Homeostasis by Mucosal Barriers. Inflamm. Regen. 38, 5. 10.1186/s41232-018-0063-z 29619131 PMC5879757

[B53] Okumura R. Takeda K. (2017). Roles of Intestinal Epithelial Cells in the Maintenance of Gut Homeostasis. Exp. Mol. Med. 49 (5), e338. 10.1038/emm.2017.20 28546564 PMC5454438

[B54] Plichta D. R. Graham D. B. Subramanian S. Xavier R. J. (2019). Therapeutic Opportunities in Inflammatory Bowel Disease: Mechanistic Dissection of Host-Microbiome Relationships. Cell 178 (5), 1041–1056. 10.1016/j.cell.2019.07.045 31442399 PMC6778965

[B55] Ren X. Zhang L. Ma X. Li J. Lu Z. (2022). Integrated Bioinformatics and Experiments Reveal the Roles and Driving Forces for HSF1 in Colorectal Cancer. Bioengineered 13 (2), 2536–2552. 10.1080/21655979.2021.2018235 35006040 PMC8974194

[B56] Ricciuto A. Griffiths A. M. (2019). Clinical Value of Fecal Calprotectin. Crit. Rev. Clin. Lab. Sci. 56 (5), 307–320. 10.1080/10408363.2019.1619159 31088326

[B57] Rodolico V. Tomasello G. Zerilli M. Martorana A. Pitruzzella A. Gammazza A. M. (2010). Hsp60 and Hsp10 Increase in Colon Mucosa of Crohn’s Disease and Ulcerative Colitis. Cell Stress Chaperones 15 (6), 877–884. 10.1007/s12192-010-0196-8 20390473 PMC3024080

[B58] Rogler G. (2014). Chronic Ulcerative Colitis and Colorectal Cancer. Cancer Lett. 345 (2), 235–241. 10.1016/j.canlet.2013.07.032 23941831

[B59] Saber S. El-Kader E. M. A. (2021). Novel Complementary Coloprotective Effects of Metformin and MCC950 by Modulating HSP90/NLRP3 Interaction and Inducing Autophagy in Rats. Inflammopharmacology 29 (1), 237–251. 10.1007/s10787-020-00730-6 32594364

[B60] Samoila I. Dinescu S. Costache M. (2020). Interplay between Cellular and Molecular Mechanisms Underlying Inflammatory Bowel Diseases Development-A Focus on Ulcerative Colitis. Cells 9 (7). 10.3390/cells9071647 PMC7408467 32659925

[B61] Shang L. Wang L. Shi X. Wang N. Zhao L. Wang J. (2020). HMGB1 Was Negatively Regulated by HSF1 and Mediated the TLR4/MyD88/NF-Κb Signal Pathway in Asthma. Life Sci. 241, 117120. 10.1016/j.lfs.2019.117120 31825792

[B62] Singh S. Fumery M. Sandborn W. J. Murad M. H. (2018). Systematic Review with Network Meta-Analysis: First- and Second-Line Pharmacotherapy for Moderate-Severe Ulcerative Colitis. Aliment. Pharmacol. Ther. 47 (2), 162–175. 10.1111/apt.14422 29205406

[B63] Soderholm A. T. Pedicord V. A. (2019). Intestinal Epithelial Cells: at the Interface of the Microbiota and Mucosal Immunity. Immunology 158 (4), 267–280. 10.1111/imm.13117 31509239 PMC6856932

[B64] Song Y. Yang H. Lin R. Jiang K. Wang B. M. (2019). The Role of Ferroptosis in Digestive System Cancer. Oncol. Lett. 18 (3), 2159–2164. 10.3892/ol.2019.10568 31402933 PMC6676710

[B65] Tanaka K. Namba T. Arai Y. Fujimoto M. Adachi H. Sobue G. (2007). Genetic Evidence for a Protective Role for Heat Shock Factor 1 and Heat Shock Protein 70 against Colitis. J. Biol. Chem. 282 (32), 23240–23252. 10.1074/jbc.M704081200 17556362

[B66] Tomasello G. Sciumé C. Rappa F. Rodolico V. Zerilli M. Martorana A. (2011). Hsp10, Hsp70, and Hsp90 Immunohistochemical Levels Change in Ulcerative Colitis after Therapy. Eur. J. Histochem 55 (4), e38. 10.4081/ejh.2011.e38 22297444 PMC3284240

[B67] Ungaro R. Colombel J. F. Lissoos T. Peyrin-Biroulet L. (2019). A Treat-To-Target Update in Ulcerative Colitis: A Systematic Review. Am. J. Gastroenterol. 114 (6), 874–883. 10.14309/ajg.0000000000000183 30908297 PMC6553548

[B68] Wang W. Zhang F. Li X. Luo J. Sun Y. Wu J. (2020). Heat Shock Transcription Factor 2 Inhibits Intestinal Epithelial Cell Apoptosis through the Mitochondrial Pathway in Ulcerative Colitis. Biochem. Biophys. Res. Commun. 527 (1), 173–179. 10.1016/j.bbrc.2020.04.103 32446363

[B69] Wang Y. Lin F. Zhu X. Leone V. A. Dalal S. Tao Y. (2018). Distinct Roles of Intracellular Heat Shock Protein 70 in Maintaining Gastrointestinal Homeostasis. Am. J. Physiol. Gastrointest. Liver Physiol. 314 (2), G164–G178. 10.1152/ajpgi.00208.2017 29051186 PMC5866418

[B70] Wells J. M. Brummer R. J. Derrien M. MacDonald T. T. Troost F. Cani P. D. (2017). Homeostasis of the Gut Barrier and Potential Biomarkers. Am. J. Physiol. Gastrointest. Liver Physiol. 312 (3), G171–G193. 10.1152/ajpgi.00048.2015 27908847 PMC5440615

[B71] Wen Y. Niu J. Zhang F. Wu J. Li M. Sun Y. (2020). Heat Shock Transcription Factor 2 Predicts Mucosal Healing and Promotes Mucosal Repair of Ulcerative Colitis. Scand. J. Gastroenterol. 55 (6), 677–686. 10.1080/00365521.2020.1774924 32538201

[B72] Wilkerson D. C. Skaggs H. S. Sarge K. D. (2007). HSF2 Binds to the Hsp90, Hsp27, and C-Fos Promoters Constitutively and Modulates Their Expression. Cell Stress Chaperones 12 (3), 283–290. 10.1379/csc-250.1 17915561 PMC1971238

[B73] Wu L. Hu C. Huang M. Jiang M. Lu L. Tang J. (2013). Heat Shock Transcription Factor 1 Attenuates TNFα-Induced Cardiomyocyte Death through Suppression of NFκB Pathway. Gene 527 (1), 89–94. 10.1016/j.gene.2013.05.024 23769970

[B74] Xie Y. Hou W. Song X. Yu Y. Huang J. Sun X. (2016). Ferroptosis: Process and Function. Cell Death Differ. 23 (3), 369–379. 10.1038/cdd.2015.158 26794443 PMC5072448

[B75] Xu M. Tao J. Yang Y. Tan S. Liu H. Jiang J. (2020). Ferroptosis Involves in Intestinal Epithelial Cell Death in Ulcerative Colitis. Cell Death Dis. 11 (2), 86. 10.1038/s41419-020-2299-1 32015337 PMC6997394

[B76] Xu Y. M. Huang D. Y. Chiu J. F. Lau A. T. (2012). Post-translational Modification of Human Heat Shock Factors and Their Functions: a Recent Update by Proteomic Approach. J. Proteome Res. 11 (5), 2625–2634. 10.1021/pr201151a 22494029

[B77] Yang Y. Jin L. Zhang J. Wang J. Zhao X. Wu G. (2017). High HSF4 Expression Is an Independent Indicator of Poor Overall Survival and Recurrence Free Survival in Patients with Primary Colorectal Cancer. IUBMB Life 69 (12), 956–961. 10.1002/iub.1692 29131521

[B78] Yashiro M. (2014). Ulcerative Colitis-Associated Colorectal Cancer. World J. Gastroenterol. 20 (44), 16389–16397. 10.3748/wjg.v20.i44.16389 25469007 PMC4248182

[B79] Zhang F. Wang W. Niu J. Yang G. Luo J. Lan D. (2020). Heat-shock Transcription Factor 2 Promotes Sodium Butyrate-Induced Autophagy by Inhibiting mTOR in Ulcerative Colitis. Exp. Cell Res. 388 (1), 111820. 10.1016/j.yexcr.2020.111820 31923427

[B80] Zhang F. Zhao W. Zhou J. Wang W. Luo J. Feng Y. (2021). Heat Shock Transcription Factor 2 Reduces the Secretion of IL-1β by Inhibiting NLRP3 Inflammasome Activation in Ulcerative Colitis. Gene 768, 145299. 10.1016/j.gene.2020.145299 33181254

[B81] Zhang Y. Wang X. Wang S. Yan Z. Li C. Zheng Y. (2020). Heat Shock Protein 27 Regulates the Inflammatory Response of Intestinal Epithelial Cells by the Nuclear Factor-Κb Pathway. Dig. Dis. Sci. 65 (12), 3514–3520. 10.1007/s10620-020-06074-z 32078087

[B82] Zhou Z. Li X. Qian Y. Liu C. Huang X. Fu M. (2020). Heat Shock Protein 90 Inhibitors Suppress Pyroptosis in THP-1 Cells. Biochem. J. 477 (20), 3923–3934. 10.1042/BCJ20200351 32497199 PMC7773284

[B83] Zininga T. Ramatsui L. Shonhai A. (2018). Heat Shock Proteins as Immunomodulants. Molecules 23 (11). 10.3390/molecules23112846 PMC6278532 30388847

